# Validation of immunomodulatory effects of lipopolysaccharide through expression profiling of Th1 and Th2 biased genes in Newcastle disease virus vaccinated indigenous chicken

**DOI:** 10.14202/vetworld.2018.437-445

**Published:** 2018-04-09

**Authors:** Rabia Bhardwaj, Ramneek Verma, Dipak Deka, P. P. Dubey, J. S. Arora, R. S. Sethi, T. C. Tolenkhomba, C. S. Mukhopadhyay

**Affiliations:** 1 School of Animal Biotechnology, Guru Angad Dev Veterinary and Animal Sciences University, Ludhiana - 141 001, Punjab, India; 2 Department of Animal Genetics and Breeding, Guru Angad Dev Veterinary and Animal Sciences University, Ludhiana - 141 001, Punjab, India; 3 Department of Animal Genetics and Breeding, College of Veterinary Science and Animal Husbandry, Central Agricultural University, Mizoram, India

**Keywords:** adjuvants, Aseel, lipopolysaccharide, Newcastle disease, vaccine

## Abstract

**Background and Aim:**

Newcastle disease (ND) is considered one of the most important poultry diseases with chicken morbidity and mortality rates up to 100%. Current vaccination programs allow the use of live attenuated vaccines in the field to protect against the disease, which alone is inefficient and requires repeat booster doses. Toll-like receptor agonists (e.g., lipopolysaccharide [LPS]) as adjuvants are the ones, most extensively studied and have shown to be very promising in delivering a robust balanced immune response. In the present study, we have evaluated the potential of LPS to elicit a strong immune response with respect to the elicitation of both Th1 (cell-mediated) and Th2 (humoral) immune arms.

**Materials and Methods:**

A total of 72 apparently healthy 1-day-old indigenous unvaccinated chicks were randomly divided into six experimental Groups A to F (n=12). At 8-week of age chicks in Group A, C, and E were vaccinated with live attenuated La Sota strain ND vaccine along with LPS, bovine serum albumin, and normal saline solution, respectively, and those in Group B, D, and E were kept separately without vaccination. Sampling was done on days 0, 1, 3, 7, 14, 21, 35, and 60 after vaccination. After vaccination and respective adjuvant application, Th1 and Th2 cytokine expression were measured in mRNA of both blood and tissue samples.

**Results:**

The results were validated by, hemagglutination inhibition and enzyme-linked immunosorbent assay tests, to check for the humoral as well as cell-mediated immune response in blood serum levels. The results showed an increase in mRNA expression of the Th1 biased cytokines in Group A (LPS+NDV) as compared to the control groups. Similar mRNA expression pattern was seen in blood as well as tissue samples. Validation of results also indicates an increase in Cell-mediated Immunity as well as a humoral immune response in Group A (LPS+NDV).

**Conclusion:**

The results of the study provided enough evidence to consider LPS as a potential vaccine adjuvants candidate against ND in chicken.

## Introduction

Host defense against invading microbial pathogens is elicited by the immune system which consists of two components: Innate and acquired immunity. To date, the main research interest in the field of immunology has been confined to the acquired immunity to analyze the mechanisms by which the antigen receptors recognize foreign antigens and exercise the response accordingly (namely, diversity and memory of adaptive immunity). The non-specific, innate immune system that lays the foundation of the overall immunological processes outranks the acquired immunity due to its wide range action against all sorts of invading pathogens as well as dictating the correct acquired immune response. However, till date, the former has not been well studied with respect to its response to different vaccine adjuvants [1]. Recent development in knowledge on the functioning of innate immunity has made the toll-like receptor (TLR)-based adjuvants to emerge as one of the promising candidates in representing a physical link between the innate and adaptive immunity. Lipopolysaccharide (LPS), an innate immune response element and also the best-characterized sensor of TLR4, is responsible for the inflammatory response and other important events required for the initiation of innate immune responses and also a potent elicitor of Th1 biased immune response [2]. A Viral disease like Newcastle disease (ND) (or Ranikhet) causes huge economic losses to the poultry sector all over the world and must be headed with immediate concern. Therefore, vaccines providing strong cell-mediated immune response apart from humoral response are required [3].

Currently available ND virus (NDV) vaccines can provide the necessary humoral immune response boost but lack considerably in providing a cell-mediated response. TLR-ligand plays a crucial role here as they combat infectious diseases by providing both inflammatory and antigen-specific immune responses [4]. Thus, LPS has come up as a useful tool in the immunomodulatory studies against NDV as it provides a rapid action force required for immediate action. Various studies show the immunomodulatory potential of LPS as adjuvants in mice models, but studies on the use of LPS as a potential immunomodulator in combination with ND vaccine in live animal experimentation are not available. An adjuvant which is a constituent of vaccine is an immunological agent which modifies the immune response by acting as a depot that provides a slow and prolonged release of antigen. The traditionally used adjuvants (e.g., aluminum salts, microbial components, synthetic polymers, emulsions, and complex surface-active compounds) are relatively toxic, suffer from weak adjuvant activity, sometimes causes severe inflammatory reactions at the site of injection and also fail to induce a strong Th1 biased (cell-mediated) immune response which limits their use. This limitation mentioned above particularly necessitates the development of novel adjuvant systems, which can elicit a strong directed immune response.

The present study evaluates the immunomodulatory potential of LPS in combination with *La Sota* vaccine in specific pathogen-free chicken by studying the mRNA expression of various Th1 and Th2 biased cytokines in both blood and tissue samples. Further, validation of the results was done by studying their serum level concentrations using ELISA, to find out as to which arm of immune response is favored.

The research plan was designed in a way to minimize the concept of booster dosages by evaluating the maximum potential of the adjuvant under study. The results obtained, contrary to the current practices, provide a valid proof of the use of adjuvant only once along with the NDV vaccine to provide a heightened as well as long lasting Th1 and Th2 biased immune response. To the best of our knowledge, this is the first ever report of the evaluation of LPS as vaccine adjuvant against ND in indigenous chicken model *per se*.

## Materials and Methods

### Ethical approval

Ethical approval for the study was obtained from Institutional Animal Ethics Committee (IAEC) (IAEC/2015/30-63; Dated: 26/02/2015).

### Experimental birds

1-day-old indigenous (Aseel) unvaccinated chicks (n=72) were procured from the Hatchery Punjab Agricultural University and were maintained in the GADVASU University Poultry Farm (Ludhiana, Punjab, India). All the chicks were kept in a separate shed, in best possible clean and disease free environment as per the available resources.

### Experimental design

A total of 72 1-day-old unvaccinated birds were maintained till 8-week of age before the start of the study and were allotted to six experimental groups (-A to -F) (Table-1). Intraocular *La Sota* strain vaccine (Ludhiana Feed Mills) was given to 8-week birds from Groups A, C, and E on day 1 of the experiment. Non-vaccinated birds were kept separate from the vaccinated birds. 100 µl of 2 mg/kg body weight of LPS (Sigma-Aldrich, Germany) was administered intravenously to the treatment group (Group-A and -B) birds (n=24) on day 1. LPS was given intravenously as it is the most efficient method for LPS injection to elicit the maximum innate immune response. Bovine serum albumin (BSA) (Group-C and -D) which served as the positive control and normal saline solution (NSS) to control group (Group-E and -F) were also injected, respectively. Peripheral blood samples (0.5 ml) were aseptically collected from the birds at day 0, 1, 3, 7, 14, 21, 35, and 60 posts 1 time vaccine and adjuvants administration. Spleen and cecal tonsils were collected in RNA later solution (Qiagen, CA, USA) at day 21, after slaughtering half of the birds. Blood and tissue samples were immediately stored at −80°C until use.

**Table-1:** Detail of experimental groups, number of experimental birds and description of each group.

Serial number	Number of Birds	Treatment
I treatment group	24	GroupA: 12 birds were vaccinated with intraocular NDV vaccine (*La Sota* strain) along with LPS administered through intravenous route in a volume of 100 μl(at 2 mg/kg body weight
		GroupB: 12 birds were administered with LPS only(no NDV)
II positive control	24	GroupC: 12 birds were given intravenous BSA(at 40 mg/kg body weight) and along with intraocular NDV vaccine (*La Sota* strain)
		GroupD: 12 birds were given BSA only(no NDV)
III Negative control	24	GroupE: 12 birds were given intravenous normal saline and vaccinated with intraocular NDV vaccine (*La Sota* strain)
		GroupF: 12 birds were given Normal saline only(no NDV)

NDV=Newcastle disease virus, LPS=Lipopolysaccharide, BSA=Bovine serum albumin

### Isolation of total RNA and preparation of complementary DNA (cDNA)

Blood and tissue samples were processed for total RNA isolation using Trizol (Ambion, USA) as per the manufacturer’s protocol and the cDNA was synthesized from total high capacity reverse transcriptase kit (Applied Biosystem, USA), following manufacturer’s instructions.

### Real-time polymerase chain reaction (PCR) quantification

Expression levels of mRNA of TLR4, interleukin-12 (IL-12), interferon gamma (IFN-γ), IL-4, and IL-5 were analyzed by real-time PCR (Bio-Rad, CA, USA) using the TaqMan Assay by design (Applied Biosystems, Life Technologies, USA) using custom designed gene-specific primers (Table-2). Chicken β-actin was used as the reference gene, and real-time PCR was performed in duplicates. Expression levels of the target genes were calculated relative to the expression of the β-actin gene and analysis was done using the ΔΔCt method [5].

**Table-2:** NCBI nucleotide accession numbers of the mRNA sequences used for custom designing and synthesis of TaqMan assay by design primers and probe^$^.

No.	Gene	Accession number
1	TLR4	KF697090.1
2	IL -4	GU119892.1
3	IL -5	AB618613
4	IFN -γ	AF424744
5	Chicken betaactin	L08165.1

$ Primer sequences are company’s copyright and thus cannot be mentioned, TLR4=Tolllike receptor 4, IL=Interleukin, IFNγ=Interferon gamma

### Evaluation of humoral immune response

Blood was collected from all the birds and serum was separated and stored at −20°C until use. Serum samples were analyzed by hemagglutination inhibition (HI) test using 1% chicken red blood cells (RBCs) according to the Office International des Epizooties recommended protocol [6]. The HI titer was determined as the highest dilution of serum sample that inhibited NDV agglutination of chicken RBCs. The antibody titer of each sample was calculated by logarithmic transformation (base 2) of the obtained titer.

### Evaluation of cellular immune response

Serum protein concentration of IFN-γ cytokine was measured in serum samples using the commercially available ELISA kit (Cusabio, GenxBio, China) according to the manufacturer’s instructions.

### Statistical analysis

Each experiment was designed to have biological and technical replicates, and representative data from that experiment was further utilized for the analysis. Three samples were pooled to make one biological replicate and experiment was done using two technical replicates. Data were analyzed using the statistical software SYSTAT (version 12.0). ANOVA and *post-hoc* analysis of the dCt values were performed using Tukey’s test to determine the significant difference in the fold change values. ELISA results were analyzed using the Curveexpert 4-PL logistic curve, using My Assay, online software (https://www.myassays.com/four-parameter-logistic-curve.assay). GraphPad Prism Evaluation version 7.0 was used for generating the graphs (https://www.graphpad.com/scientific-software/prism/).

## Results

### mRNA expression of immune response genes in whole blood

#### Th1-biased cytokine genes

**Figure-1:**
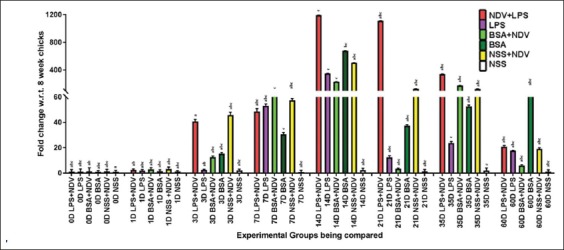
Relative expression of interleukin (IL)-12 cytokine gene in whole blood of indigenous chicken over a period of 60 days. A significant difference in the fold change (p<0.05) is shown with different symbols, determined by *post-hoc* analysis test of the dCt values of the IL-12 gene under experiment. Each bar indicates a mean±standard error of a representative experiment.

IL-12 and IFN-γ genes’ expression was ascertained at various days’ interval after intravenous injection of LPS and NDV. There was a significant increase (p<0.05) in IL-12 on day 14 and 21, till day 35 in both Group-A (LPS+NDV) and -B (LPS). There was a rise in IL-12 expression in Group-C (BSA+NDV) and Group-D (BSA) on day 14 but with no significant difference (p<0.05) between the vaccinated (Group-C) and the non-vaccinated (Group-D) groups (Figure-1). The expression pattern of Group-E (NDV), however, showed a rise in IL-12 fold change at day 14 only. There was no significant (p<0.05) increase observed in Group-F (NSS) for IL-12 expression. The result signifies an upregulation of IL-12 genes in LPS injected groups, with maximum fold change values observed in Group-A (LPS+NDV).

**Figure-2:**
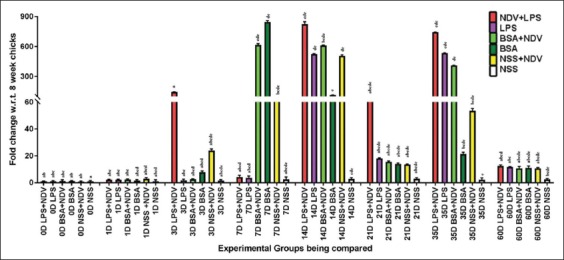
Relative expression of interferon-gamma (IFN-γ) cytokine gene in whole blood of indigenous chicken over a period of 60 days. A significant difference in the fold change (p<0.05) is shown with different symbols, determined by *post-hoc* analysis test of the dCt values of the IFN-γ gene under experiment. Each bar indicates a mean±standard error of a representative experiment.

The expression of IFN-γ gene was also maximum in Group-A (LPS+NDV) and -B (LPS) at day 14. There was a decline in IFN-γ fold change on day 21, which rose again at day 35 (Figure-2). The expression of IFN-γ gene in Group-C (BSA+NDV) and -D (BSA) showed a rise at day 7 and 14, declining rapidly after that. The expression pattern of IFN-γ in Group E (NDV) was very similar to that of IL-12 with significant (p<0.05) rise in IFN-γ gene expression only day14. No significant increase in IL-12 expression of Group-F (NSS) was observed.

#### Th2-biased cytokine genes

**Figure-3:**
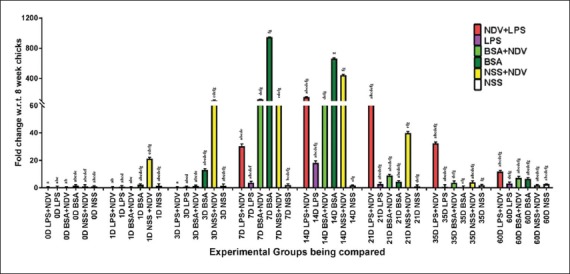
Relative expression of interleukin (IL)-4 cytokine gene in whole blood of indigenous chicken over a period of 60 days. A significant difference in the fold change (p<0.05) is shown with different symbols, determined by *post-hoc* analysis test of the dCt values of the IL-4 gene under experiment. Each bar indicates a mean±standard error of a representative experiment.

The expression of IL-4 is observed maximum in NDV vaccinated groups. A sudden increase in fold change was observed in Group-E (NDV) on day 3 and day 14. The maximum increase in IL-4 gene expression was observed in Group-D (BSA) on day 7. The sudden increase in fold change continued until day 14, which declined rapidly. Group-A, on the other hand, showed a significant (p<0.05) rise in IL-4 gene expression only at day 14 (Figure-3).

The real-time TaqMan analysis of ChIL-5 gene revealed an absence of expression in all the groups, at all the days. The samples were run in triplicates, and β-actin (housekeeping gene) expression double confirmed the cDNA samples. Later, it was confirmed by another literature search that Ch IL-5 is a pseudogene and it is not expressed at mRNA level [7].

#### Expression of TLR4 gene

**Figure-4:**
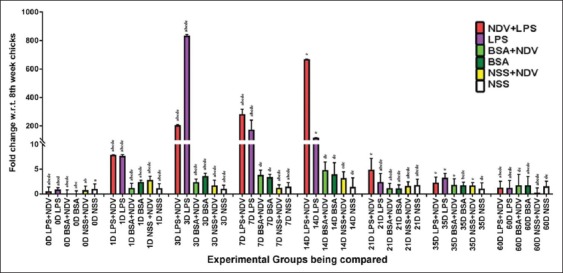
Relative expression of toll-like receptor 4 (TLR4) cytokine genes in whole blood of indigenous chicken over 60 days period. A significant difference in the fold change (p<0.05) is shown with different symbols, determined by *post-hoc* analysis test of the dCt values of the TLR4 gene under experiment. Each bar indicates a mean±standard error of a representative experiment.

TLR4 is the best-characterized sensor of LPS; therefore, its expression was also checked at mRNA level. The significant (p<0.05) gene expression of TLR4 gene was only observed in LPS injected groups, i.e., Group-A (LPS+NDV) and Group-B (LPS). The expression pattern observed in Figure-4 shows an increase in gene expression on day 3 and 7, for both Group-A and -B. The fold change later declined rapidly after day 14.

#### mRNA expression of immune response genes in spleen

**Figure-5:**
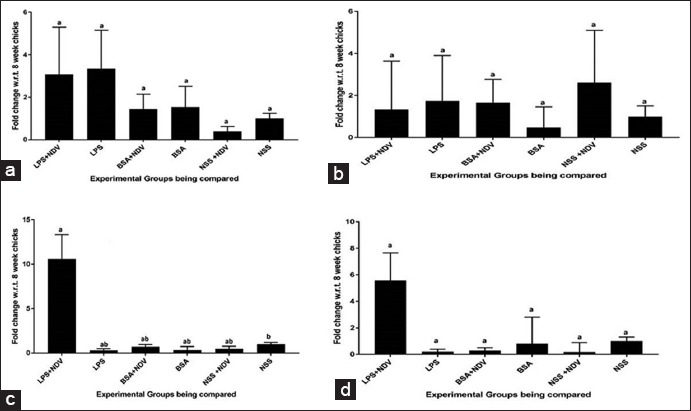
Relative expressions of cytokine genes in 21 days spleen tissue samples; (a) toll-like receptor-4, (b) interleukin (IL)-4, (c) IL-12, (d) interferon gamma. Each bar indicates a mean±standard error of a representative experiment.

The maximum fold change for both IL-12 and IFN-γ gene expression was seen in Group-A (LPS+NDV). Figure-5c and d shows an absence of IL-12 and IFN-γ expression in rest of the groups. There was a significant (p<0.05) increase in IL-4 gene expression in Group-E (NDV) as compared to all the other groups in 21-day spleen samples (Figure-5b) and the expression pattern was very similar to that of what was observed in whole blood. There was no significant increase in TLR4 gene expression in any of the groups (Figure-5a). IL-5 gene expression was also absent in spleen.

#### mRNA expression of immune response genes in cecal tonsil

**Figure-6:**
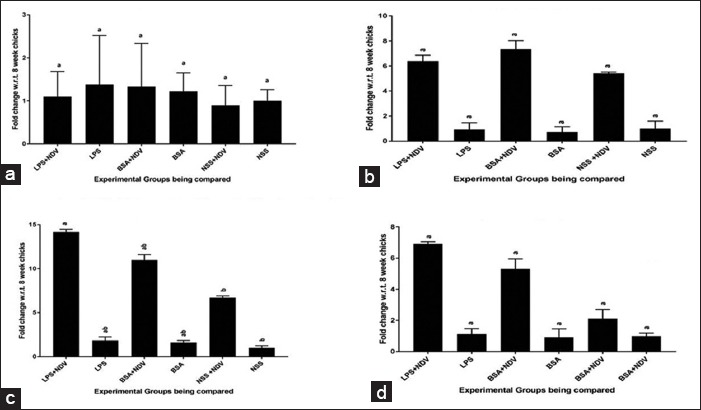
Relative expressions of cytokine genes in 21 days in cecal tonsil tissue samples; (a) toll-like receptor 4, (b) interleukin-4 (IL-4), (c) IL-12, (d) interferon gamma. Each bar indicates a mean±standard error of a representative experiment

The gene expression pattern of immune response gene in cecal tonsils predicts a significant increase in IL-12, IFN-γ, and IL-4 gene expression in NDV vaccinated groups, namely, Group-A, -C, and -E with maximum fold change observed in Group-A (LPS+NDV) for both IL-12 and IFN-γ genes (Figure-6c and d). There was no significant increase in IL-4 and TLR4 gene expression in all the groups (Figure-6a and b). IL-5 gene expression was absent in cecal tonsils too.

#### Humoral immune response

**Figure-7:**
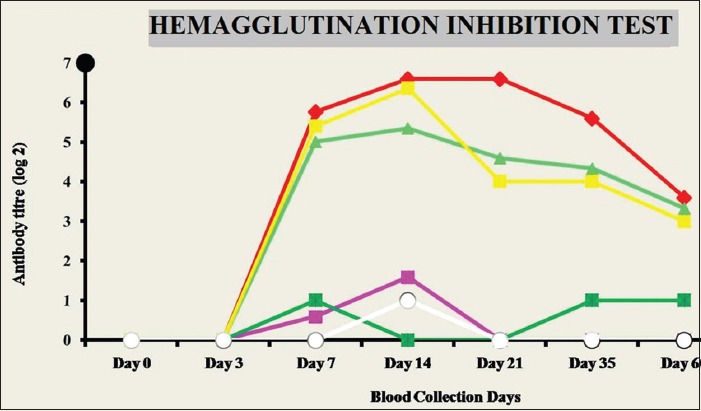
Hemagglutination inhibition test on antibody titer (log 2) expressed as mean in the sera of experimental indigenous specific pathogen-free chicken on 60 days experimental samples.

The humoral immune response was monitored by hemagglutination inhibition (HI) test. The mean of the antibody titer was used to estimate the log2 HI titers (Figure-7). The result obtained depicts the absence of HI titer on day 0 and 3. Furthermore, the HI titer values were nil in unvaccinated groups (Group-B, -D, and -F). A clear view of the HI titer graph showed the highest titer values in Group-A (LPS+NDV) at all points during the entire collection schedule. Group-E (NDV) also showed a higher titer value on day 7 but the values later declined rapidly.

#### Cell-mediated immune response

**Figure-8:**
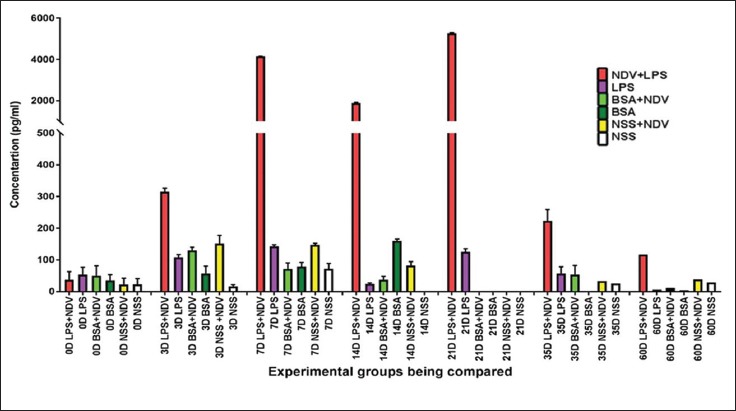
Enzyme-linked immunosorbent assay test was done in the 8-week experimental specific pathogen-free indigenous chicken, and serum protein concentration (pg/ml) was calculated by 4-PL logistic curve analysis of the O.D. value. Three independent experiments were done, and data of a representative experiment are presented. Each bar represents a mean±standard error.

The cell-mediated immune response was assessed by ELISA to find out the serum level protein concentration of the IFN-γ (Th1 biased cytokine) (Figure-8). The IFN-γ serum protein concentration validates the mRNA level expression of IFN-γ. The treatment Group-A (LPS+NDV) has the maximum protein concentration at all days. The serum protein concentration was also observed on day 0 (before vaccination) as some basal level protein concentration as IFN-γ is normally found in blood serum. There was a rapid increase in protein concentration of IFN-γ on day 3. The protein concentration values spiked maximally in Group-A (LPS+NDV), and the pattern continued for all collection days. The maximum protein concentration was observed on day 21 which declined after that. The values of IFN-γ protein concentration at day 3, 7, 14, 21, 35, and 60 are 314.3±11.9, 4146.6±7.1, 1883.5±35.4, 5268.3±27.5, 223±36, and 117 pg/ml, respectively.

## Discussion

Our current study evaluates the adjuvant potential of LPS was in 8 weeks unvaccinated Aseel chicken by monitoring the mRNA gene expression as well as serum protein concentration of immune response genes, namely, IL-12, IFN-γ, and IL-4. Till date, no such report of similar experimentation in indigenous chicken has been documented. In Avians, the adjuvant potential of ligands such as CpG oligonucleotides against NDV as well as avian influenza virus has been well documented in layers and another commercial flock [8,9]. Furthermore, LPS is a popular candidate for vaccine adjuvants in other non-avian models like mice [10]. However, the study of the effect of adjuvants in the indigenous chicken is still lacking, with almost no reports of LPS being used as an effective vaccine adjuvant in chicken models. The results obtained from our study shows a significant upregulation of both TLR4 (LPS sensor) as well as other cytokine genes (IL-12, IFN-γ, and IL-4) in both blood and tissue samples (spleen and cecal tonsils). A similar study evaluating the adjuvant potential of resiquimod (TLR7 agonist), against ND vaccine, was done which was in complete agreement to our current research work showing a similar expression pattern in IFN-γ and IL-4 cytokines in spleen [11].

Our study conducted an evaluation of the importance of LPS along with live-attenuated *La Sota* strain vaccine, which predicts the excess of Th1 biased immunity, out of the two arms of the immune response. The results obtained showed an increase in expression of Th1 biased cytokine genes (IL-12 and IFN-γ). Furthermore, the Th2 biased immune arm was significantly (p<0.05) altered after positive stimulation by both adjuvant and vaccine combination. Although IL-4 gene expression was limited in Group-A (LPS+NDV) because of the presence of LPS, which is a potent Th1 elicitor as it directs the naïve helper cells (Th0) toward the Th1 biased. Taylor *et al*. [10], conducted the study demonstrating the importance of LPS as a vaccine adjuvant as it promotes the Th1 biased immunity through recruitment of naïve T helper cells toward Th1 biased response. The absence of LPS from the vaccine results in strong Th2 biased immunity with significantly more IL-4 than IFN-gamma. Therefore, from the mRNA expression studies, we can deduce that LPS is responsible for eliciting the Th1 biased immune response but also a heightened response is seen when LPS is given in combination with NDV vaccine. The groups without NDV vaccine (Group-B, -D, and -F), showed no significant increase in either the mRNA gene expression as well as serum protein concentration of any of the cytokine under study.

The comparison of the mRNA level gene expression and serum protein concentration of IFN-γ provides an insight of the molecular machinery working after adjuvant and vaccine administration. The IFN-γ serum estimation depicts the rise of the IFN-γ at both the mRNA as well as at the serum level. There was an increase in both mRNA fold change as well as serum protein concentration on day 3 in treatment Group-A (LPS+NDV). Later, a decline in mRNA expression was seen in Group-A on day 7 and day 21. The serum IFN-γ value provides a relevant explanation for the drop in mRNA expression levels. The excess amount of IFN-γ serum concentration causes a negative feedback type of signaling response which causes a temporary shutdown of the mRNA machinery for the IFN-γ gene. The mRNA synthesis resumes as soon as the serum level concentration is reached back to normal level. The clear correlation seen in the protein and mRNA levels of IFN-γ can be certainly backed up by a similar study of comparison of protein abundance and mRNA expression levels on a genomic scale. The study provides validation of our current hypothesis of the possibility of some correlation between the level of mRNA and that of the protein [12]. Thus, we can clearly validate our mRNA expression study with that of serum protein concentration.

The main objective of our current experimental plan was to find the best possible adjuvant and vaccine combination to minimize the booster dosage as well as maximize the immune response. The fold change increase in both Th1 and Th2 biased cytokine expression predicts the elicitation of both cell-mediated as well as a humoral immune response in treatment groups (Group-A and -B) with only a single dosage of adjuvant and vaccine combination. The use of LPS to reduce the vaccine dosage as well to incorporate the cellular immunity proved out to be a dual advantage. The results obtained by our study were favored by a similar study which used Monatanide™ for cellular immune response in poultry and also emphasized on its capability to increase the cellular immune response that could help to extend the cross-protection against different viral strains or serotypes with reduction of injection doses of inactivated ND poultry vaccines [13].

Furthermore, there was a greater inclination toward Th1 biased immunity as compared to that of Th2 biased, as LPS, as discussed above, is a strong Th1 elicitor. The cell-mediated immunity has an advantage over the humoral immune response as it provides better protection as well as clearance of intracellular pathogens like viruses. Therefore, an adjuvant which provides a strong cell-mediated immune response is beneficial for viral diseases like ND. The results are in complete agreement with another study of *H. pylori* challenge in mice which depicts that Th1 biased immunity is responsible for providing a stronger systemic as well local immune response [14]. Another, usage of bacterial cell-wall component for vaccine potentiation of the viral disease can be seen in a similar well-known vaccine of viral origin, the live-attenuated yellow fever vaccine. It activates multiple subsets of dendritic cells through the incorporation of TLR2 agonists (bacterial cell wall components) to deliver robust immune responses [15].

LPS, when administered in combination with ND vaccine, elicits significant (p<0.05) TLR4 expression. TLR4 is an innate immune response element, protects against Gram-negative bacteria and LPS recognition has been very well studied and TLR4 is its best-characterized sensor [16]. Thus, increase in TLR4 expression can also be correlated with that of innate immune elicitation. Furthermore, the high cytokine production at mRNA and serum level predicts the elicitation of the adaptive immune arm by LPS administration. A recent study conducted by Abu-Baker and Masoud (2016) where the importance of LPS based vaccine was evaluated to provide high cross-protection against various strains of pathogen in mice, supports our hypothesis of LPS being able to deliver a combinatorial protection to both NDV and other Gram-negative bacterial infection by eliciting both innate as well as adaptive immune response when given in combination with ND vaccine [17].

## Conclusion

Administration of LPS with ND vaccine caused an upregulation of expression of IL-12, IFN-γ, IL-4, and TLR4 in both blood and tissue sample. Furthermore, the serum antibody levels and HI test validated the cytokine upregulation pattern. The adjuvant potential, thus, was confirmed by elicitation of both antigen-specific humoral and cellular immune responses only in a single dosage of vaccine and adjuvant in indigenous chicken.

## Author’s Contributions

RB, CSM, RV, and DD: Conception and design of the study. RB and CSM: Acquisition of data.RB, CSM, RV, and DD: Analysis and Interpretation of results.RB, CSM, and RV: Drafted the manuscript. RV, CSM, DD, PPD, JSA, RSS and TCT: Critical revision. All authors read and approved the final manuscript.
